# NMI inhibits cancer stem cell traits by downregulating hTERT in breast cancer

**DOI:** 10.1038/cddis.2017.200

**Published:** 2017-05-11

**Authors:** Xu Feng, Xiangdong Xu, Xiangsheng Xiao, Kun Zou, Wendan Yu, Jiali Wu, Ranran Tang, Yue Gao, Jiaojiao Hao, Xinrui Zhao, Yina Liao, Yiming Chen, Wenlin Huang, Wei Guo, Lan Kang, Wuguo Deng

**Affiliations:** 1 Institute of Cancer Stem Cell & The First Affiliated Hospital, Dalian Medical University, Dalian, China; 2 Sun Yat-sen University Cancer Center, State Key Laboratory of Oncology, Collaborative Innovation Center of Cancer Medicine, Guangzhou, China; 3 Department of Thyroid & Breast Surgery, The First Affiliated Hospital, Sun Yat-sen University, Guangzhou, China; 4 State Key Laboratory of Targeted Drug for Tumors of Guangdong Province, Guangzhou Double Bioproduct Inc., Guangzhou, China

## Abstract

N-myc and STAT interactor (NMI) has been proved to bind to different transcription factors to regulate a variety of signaling mechanisms including DNA damage, cell cycle and epithelial–mesenchymal transition. However, the role of NMI in the regulation of cancer stem cells (CSCs) remains poorly understood. In this study, we investigated the regulation of NMI on CSCs traits in breast cancer and uncovered the underlying molecular mechanisms. We found that NMI was lowly expressed in breast cancer stem cells (BCSCs)-enriched populations. Knockdown of NMI promoted CSCs traits while its overexpression inhibited CSCs traits, including the expression of CSC-related markers, the number of CD44^+^CD24^−^ cell populations and the ability of mammospheres formation. We also found that NMI-mediated regulation of BCSCs traits was at least partially realized through the modulation of hTERT signaling. NMI knockdown upregulated hTERT expression while its overexpression downregulated hTERT in breast cancer cells, and the changes in CSCs traits and cell invasion ability mediated by NMI were rescued by hTERT. The *in vivo* study also validated that NMI knockdown promoted breast cancer growth by upregulating hTERT signaling in a mouse model. Moreover, further analyses for the clinical samples demonstrated that NMI expression was negatively correlated with hTERT expression and the low NMI/high hTERT expression was associated with the worse status of clinical TNM stages in breast cancer patients. Furthermore, we demonstrated that the interaction of YY1 protein with NMI and its involvement in NMI-mediated transcriptional regulation of hTERT in breast cancer cells. Collectively, our results provide new insights into understanding the regulatory mechanism of CSCs and suggest that the NMI-YY1-hTERT signaling axis may be a potential therapeutic target for breast cancers.

Breast cancer is one of the leading mortal causes from cancer among women worldwide.^1, 2^ Surgery, radiotherapy, chemotherapy and hormone therapy are still the main and common therapeutic approaches to treat breast cancer.^3^ However, some chances to recurrence and metastasis limited their efficiencies. Cancer stem cells (CSCs), including breast cancer stem cells (BCSCs), have been shown to play important roles in cancer’s recurrence and metastasis.^4, 5, 6^ BCSCs are also relatively resistant to chemotherapy and radiotherapy compared with their non-tumorigenic progeny.^7, 8, 9^ Therefore, it is necessary to discover and identify the novel and specific molecular targets and their related signaling pathways to inhibit the mammary stem/progenitor cell population and suppress carcinogenesis and tumor metastasis.

NMI is a protein involved in the transcriptional regulation of genes. It lacks an intrinsic transcriptional activation domain, but can act as a co-activator protein to recruit a group of sequence-specific transcription factors, such as c-myc, N-myc, Sox-10 and STATs.^10, 11, 12^ NMI can be induced by IFN and mediates a variety of signaling mechanisms. Recent studies have shown that NMI can suppress tumor invasion and metastasis by inhibiting the acetylation of p65 through NF-*κ*B pathway.^13^ Loss of NMI can enhance the invasive ability of breast cancer by aberrant activation of TGF-*β*/SMAD signaling.^14^ NMI can also suppress tumor growth by inhibiting the Wnt/β-catenin signaling via upregulation of DKK1.^15^ However, whether NMI has anti-cancer role through regulating the growth of cancer stem cells (CSCs) has not been reported.

Human telomerase reverse transcriptase (hTERT) is a catalytic component of telomerase, which can elongate telomeric DNA.^16^ It is expressed in both bulk cancer cells and CSCs.^17^ Recent studies have shown that hTERT expression is closely correlated with a clinical aggressiveness and poor prognosis in many human malignancies.^18, 19, 20, 21^ In addition, hTERT has been shown to stimulate EMT and induce stemness in human gastric cancer cells, thereby promote cancer metastasis and recurrence.^22^ The hTERT inhibitor, imetelstat, has been shown to target the CSCs population in a number of tumor types, suggesting CSCs are sensitive to telomerase inhibition.^23, 24^ These findings make telomerase inhibition a striking strategy for potential cancer therapeutics by targeting CSCs.

In this study, we tried to uncover the potential function and molecular mechanisms of NMI in breast cancer, especially in BCSCs. We not only found its low expression in BCSC-enriched populations, its regulation on CSCs traits, but also found that NMI-mediated regulation of BCSCs traits was partially realized through the modulation of hTERT signaling. NMI negatively regulated hTERT expression in breast cancer cells, and hTERT silencing or overexpression reversed the NMI silencing or overexpression-mediated changes in BCSCs traits *in vitro* and *in vivo*. Moreover, the involvement of YY1 protein in NMI-mediated transcriptional regulation of hTERT in breast cancer was identified and confirmed. Combined with the analysis from the clinical samples demonstrating that NMI expression was inversely correlated with hTERT and the low NMI/high hTERT expression was associated with the worse status of clinical TNM stages in breast cancer patients, our findings may provide new insights into identifying the unknown central proteins involved in regulating BCSCs traits, understanding the underlying regulatory mechanism of such regulation and exploring new therapeutic targets for breast cancer treatment.

## Results

### NMI is downregulated in BCSCs-enriched populations

**Figure 1:**
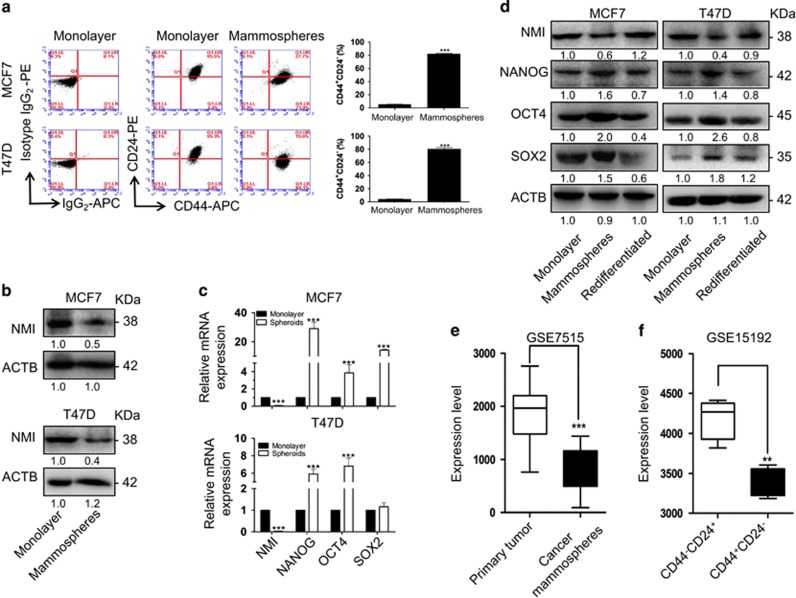
NMI is lowly expressed in breast cancer stem cell-enriched populations. (**a**) The MCF7 and T47D cells were cultured in mammosphere culture condition to enrich BCSCs. The CD44^+^CD24^−^ cells population, which represented for BCSCs in mammospheres were analyzed by FACS. The monolayer cells in the normal culture condition were used as the control. (**b**) The NMI expression level was detected by western blot and quantitatively expressing values were analyzed in mammospheres compared with monolayer cells. (**c**), The mRNA expression of NMI and cancer stem-like cell related markers Nanog Oct4 and Sox2 in monolayer and mammospheres were detected by qRT-PCR. (**d**), The protein expression of NMI and the CSC-related markers Nanog Oct4 and Sox2 in monolayer, mammospheres and redifferentiated sphere-forming cells were detected by Western blot analysis. (**e**), The NMI mRNA levels in primary breast patient tissue and cancer spheroids were analyzed using genome mRNA expression profiling data (GSE7515) from GEO database. (**f**), The NMI mRNA levels in CD44^+^CD24^−^ and CD44^−^CD24^+^ cell populations were analyzed using GEO database (GSE15192). The data were represented as the mean±S.D. of three independent experiments. The level of significance was indicated by ***P<0.001

To assess the role of NMI in BCSCs, we first determined the expression levels of NMI and CSC-related markers in mammospheres. The breast cancer cells were cultured in a suspension culture condition to enrich mammospheres. In the suspension culture condition, the CD44^+^CD24^−^ BCSCs populations were enriched (Figure 1a), and the expression of NMI and CSC-related markers in mammospheres were analyzed at protein and mRNA levels by western blot (Figures 1b and d) and qRT-PCR (Figure 1c), respectively. The results showed that the NMI was downregulated in mammospheres at both protein (Figures 1b and d) and mRNA levels (Figure 1c) as compared with the monolayer adherent culture. However, the levels of the CSC-related markers (NANOG, OCT4 and SOX2) were upregulated in mammospheres (Figures 1c and d). When the mammospheres were dissociated and cultured in the differentiation medium (RMPI1640 with 10% FBS), the level of NMI was increased but the CSC-related markers were decreased (Figure 1d). Moreover, we also analyzed the expression level of NMI using genome mRNA expression profiling data (GSE7515, GSE15192) from GEO database. As shown in Figures 1e and f, the expression of NMI was downregulated in breast cancer mammospheres compared with primary breast cancer (Figure 1e) and NMI was also downregulated in the CD44^+^CD24^−^ cells compared with the CD44^−^CD24^+^ cell populations (Figure 1f). These results indicate that NMI is downregulated in the BCSCs-enriched populations.

### NMI knockdown promotes CSCs properties of breast cancer cells

**Figure 2:**
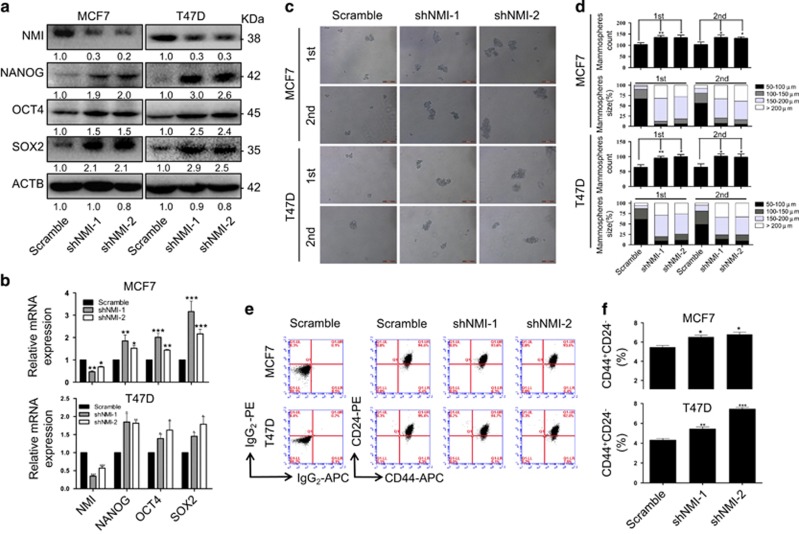
NMI knockdown promotes CSCs properties of breast cancer cells. (**a,b**) The MCF7 and T47D cells were transfected with a NMI-specific shRNA for 48 h, the expression of NMI and the CSC-related markers Nanog, Oct4, Sox2 were determined by western blot (**a**) and qRT-PCR (**b**). (**c**) The representative images of mammosphere and serially passage mammospheres formation of MCF7 and T47D cells. Scale bars, 200 *μ*m. (**d**) Quantitatively analysis of mammosphere number and size (mammosphere >50 *μ*m). (**e**) The effect of NMI knockdown on CD44^+^CD24^−^ was analyzed by FACS. (**f**) Quantitatively analysis of CD44^+^CD24^-^. The data were represented as the mean±S.D. of three independent experiments. The level of significance was indicated by *P<0.05, **P<0.01, ***P<0.001

To explore the function of NMI in BCSCs, we first analyzed the expression level of NMI in different human breast cancer cell lines by western blot and found that NMI displayed comparatively high expression in MCF7 and T47D while low expression in MDA-MB-231 (data not shown). So we chose MCF7 and T47D to knockdown NMI and MDA-MB-231 to overexpress NMI. We knocked down NMI and detected its influence on the properties of BCSCs. It showed that knockdown of NMI upregulated the expression of the CSC-related markers at both protein and mRNA levels (Figures 2a and b). Moreover, NMI knockdown also significantly increased the mammosphere number and size in both MCF7 cells and T47D cells (Figures 2c and d). Similarly, the same phenomenon was observed in the CD44^+^CD24^−^ proportion (Figures 2e and f). Recent evidence has shown that the ABC family of transporters is associated with CSCs.^25, 26, 27, 28^ Thus, we also detected their expression when NMI was knocked down. The results showed that knockdown of NMI led to the increased expression of ABC transporter genes such as ABCG2 and ABCB1 (Supplementary Figure 1). These results demonstrate that NMI acts as a potential repressor to inhibit CSCs properties of breast cancer cells.

### NMI overexpression inhibits CSCs properties of breast cancer cells

**Figure 3:**
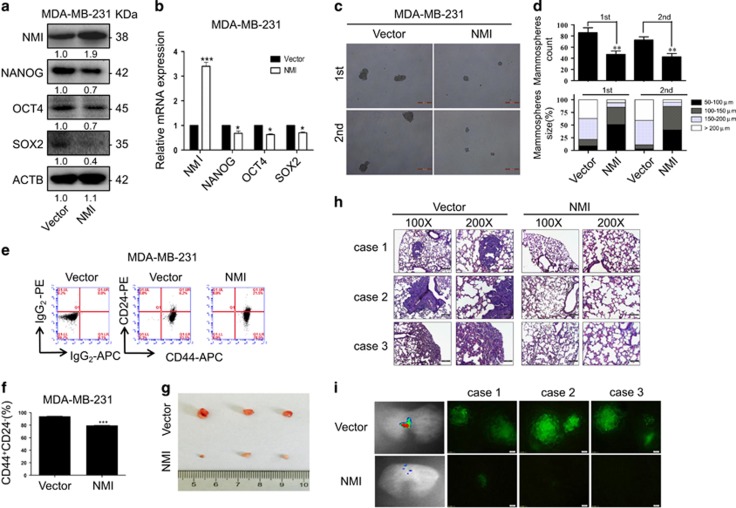
NMI overexpression inhibits CSCs properties of breast cancer cells. (**a,b**) The MDA-MB-231 cells were transfected with NMI overexpressing plasmid for 48 h, the expression of NMI and the CSC-related markers Nanog, Oct4, Sox2 were determined by western blot (**a**) and qRT-PCR (**b**). (**c**) The representative images of mammosphere and serially passage mammospheres formation of MDA-MB-231 cells. Scale bars, 200 *μ*m. (**d**) Quantitatively analysis of mammosphere number and size (mammosphere >50 *μ*m). (**e**) The effect of NMI overexpression or knockdown on CD44^+^CD24^−^ was analyzed by FACS. (**f**) Quantitatively analysis of CD44^+^CD24^−^. (**g**) The xenografts of MDA-MB-231 cells were harvested and the pictures of the tumors were obtained. (**h**) Lung metastasis was detected by H&E staining after MDA-MB-231 stable cell lines were injected into tail vein. (**i**) Lung metastasis was detected by bioluminescent imaging after GFP-labeled MDA-MB-231 stable cell lines were injected into tail vein and lung tissues were imaging by fluorescence microscope. The data were represented as the mean±S.D. of three independent experiments. The level of significance was indicated by *P<0.05, **P<0.01, ***P<0.001

Next, we analyzed the function of NMI on the properties of BCSCs by overexpressing it in MDA-MB-231 cells. Compared with the cells expressing the empty vector, the CSC-related markers of cells with NMI overexpression were decreased at both protein and mRNA level (Figures 3a and b). Moreover, NMI overexpression also significantly inhibited the mammosphere number and size in MDA-MB-231 as compared with the control group (Figures 3c and d). Similarly, the same phenomenon was observed in the CD44^+^CD24^−^ proportion (Figures 3e and f). Furthermore, the overexpression of NMI also significantly inhibited breast tumorigenicity in mouse model bearing xenografts of MDA-MB-231 cells (Figure 3g). H&E staining detection and bioluminescent imaging both showed that when MDA-MB-231 cells with NMI overexpression were injected into tail vein of mice, the lung metastasis ability was inhibited (Figures 3h and i). Hence, NMI overexpression significantly impaired CSCs expansion and tumorigenicity of breast cancer cells.

### NMI inhibits BCSCs traits by downregulating hTERT expression

**Figure 4:**
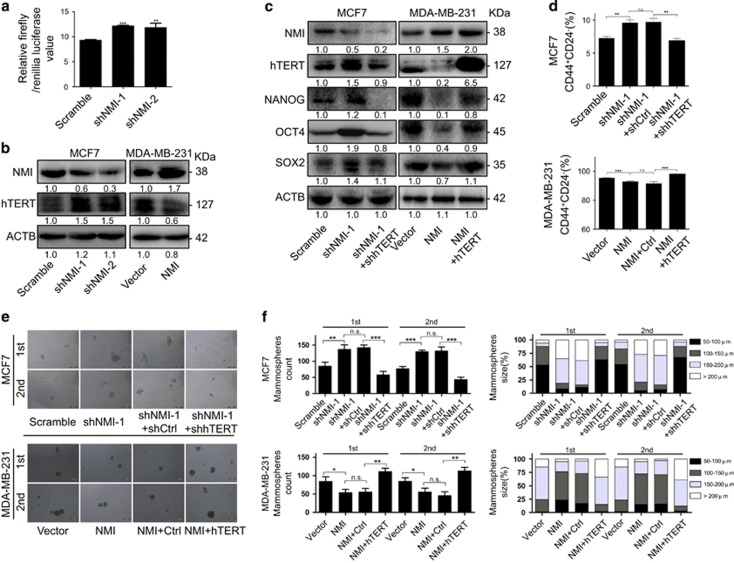
NMI inhibits CSCs traits by downregulating hTERT. (**a**) NMI knockdown by shRNA enhanced hTERT promoter-driven luciferase expression. MCF7 cells were co-transfected with the plasmids of hTERT promoter-driven luciferase and NMI shRNA or non-sense shRNA for 48 h followed by a dual luciferase assay. (**b**) MCF7 cells were transfected with NMI-specific shRNAs while MDA-MB-231 cells were transfected with NMI overexpressing plasmid for 48 h, the expression of NMI itself and hTERT were determined by western blot. (**c–f**) MCF7 cells were transfected with negative control, shNMI and shNMI/shhTERT while MDA-MB-231 cells were transfected with control vector, NMI overexpression plasmid, NMI overexpression plasmid plus hTERT overexpression plasmid for 48 h, the expression of NMI, hTERT, CSC-related markers were detected by western blot (**c**), and the CD44^+^CD24^−^ population (**d**), mammosphere and serially passage mammospheres formation ability (**e**,**f**) were also detected. The data were represented as the mean±S.D. of three independent experiments. The level of significance was indicated by *P<0.05, **P<0.01, ***P<0.001

NMI has been reported to regulate hTERT promoter activity.^29^ Based on the key role played by hTERT in CSCs,^17, 30, 31, 32^ we therefore hypothesized that hTERT might be involved in the regulation of CSCs traits mediated by NMI. To verify this, we first detected the effect of NMI on the promoter activity of hTERT. The results showed that NMI knockdown markedly upregulated the expression of luciferase driven by the hTERT promoter (Figure 4a). Additionally, NMI knockdown also upregulated the expression of hTERT protein, while its overexpression downregulated the hTERT protein levels in breast cancer cells (Figure 4b). Furthermore, we found that the expression changes of the CSC-related markers caused by NMI knockdown were rescued by hTERT knockdown, and the expression changes caused by NMI overexpression were also rescued by hTERT overexpression (Figure 4c). Similarly, knockdown of hTERT significantly impaired the improved CD44^+^CD24^−^ population ratio caused by NMI knockdown, while its overexpression reversed NMI overexpression-mediated decrease of CD44^+^CD24^−^ population ratio (Figure 4d). The enhanced formation ability both in number and size of mammospheres caused by NMI knockdown could be attenuated again by hTERT knockdown, and the NMI-mediated decrease of mammosphere number and size could be promoted again by the overexpressed hTERT (Figures 4e and f). Together, these results suggest that NMI inhibits cancer stem-like cell traits by downregulating hTERT signaling in breast cancer.

### NMI suppresses EMT in breast cancer cell lines and tumorigenicity in mouse model with xenografts of human breast cancer by downregulating hTERT

**Figure 5:**
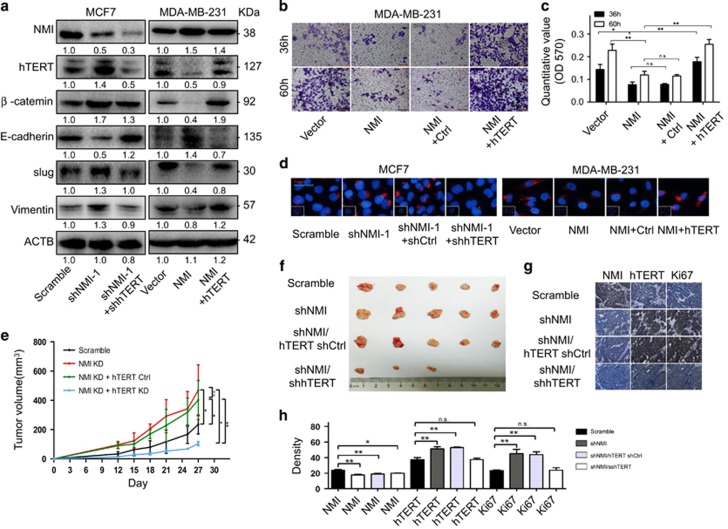
NMI suppresses EMT in breast cancer cell lines and tumorigenicity in a human breast cancer mouse model *in vivo* by downregulating hTERT. (**a–d**) MCF7 cells were transfected with negative control, shNMI and shNMI/shhTERT while MDA-MB-231 cells were transfected with control vector, NMI overexpression plasmid, NMI overexpression plasmid plus hTERT overexpression plasmid for 48 h, the expression of NMI, hTERT and EMT-related markers were detected by western blot (**a**), cell invasion ability (**b,c**) and immunofluorescence of EMT marker Vimentin (**d**) were also detected. (**e**) Tumor diameters were measured at a regular interval of 3 days for up to 27 days and the tumor volume was calculated. (**f**) The xenografts of MCF7 cells were harvested at 30 days after injection, and the pictures of the tumors were obtained. (**g**) Immunohistochemistry staining was used to detect the expression of NMI, hTERT and Ki67 (X 200). (**h**) Quantification of NMI, hTERT and Ki67 staining. The data were represented as the mean±S.D. of three independent experiments. The level of significance was indicated by *P<0.05, **P<0.01

Recent evidences have shown that EMT factors are associated with stemness in cancer cells, suggesting the existence of a link between EMT and CSCs.^33, 34^ We hereby tested the effect of the NMI/hTERT signaling on the metastasis-related properties, and found that NMI knockdown upregulated the expression of EMT-related proteins, while NMI overexpression downregulated the EMT-related proteins in breast cancer cells (Figure 5a). Moreover, consistent with the results ahead, hTERT silencing or overexpression reversed NMI-mediated expression changes of EMT-related proteins (Figure 5a). Similarly, NMI overexpression-mediated inhibition on cell invasion ability was also rescued by hTERT overexpression (Figures 5b and c). Immumofluorescence assay was further used to monitor the expression of vimentin, a metastasis-related marker, and the images indicated that NMI-mediated regulation of vimentin expression was rescued by the hTERT (Figure 5d). All the results collectively demonstrated NMI’s regulation on EMT in breast cancer cells by targeting hTERT.

We next validated the NMI-mediated regulation of tumor growth via the hTERT signaling in a human breast cancer mouse model *in vivo*. The MCF7 cells with different expression of NMI and hTERT were subcutaneously injected into nude mice. As shown in Figures 5e and f, knockdown of NMI significantly promoted tumor growth. However, inhibition of hTERT expression by hTERT shRNA effectively rescued the *in vivo* tumor progression enhanced by NMI knockdown. Notably, the mice with simultaneous knockdown of NMI and hTERT displayed a decreased tumor-initiating ability compared with the control mice without any gene knockdown. Two mice in this group even failed to form tumors, so only 3 tumors were shown in shNMI/shhTERT group. Moreover, the immunohistochemical analyses for the tumor tissues of mice revealed that NMI knockdown promoted the expression of hTERT and Ki67 *in vivo*, and such promotion was similarly reversed by hTERT knockdown (Figures 5g and h). Taken together, these data confirmed that the NMI-mediated regulation of tumor growth was realized through the modulation of hTERT signaling in breast cancer.

### YY1 interacts with NMI to mediate the downregulation of hTERT

**Figure 6:**
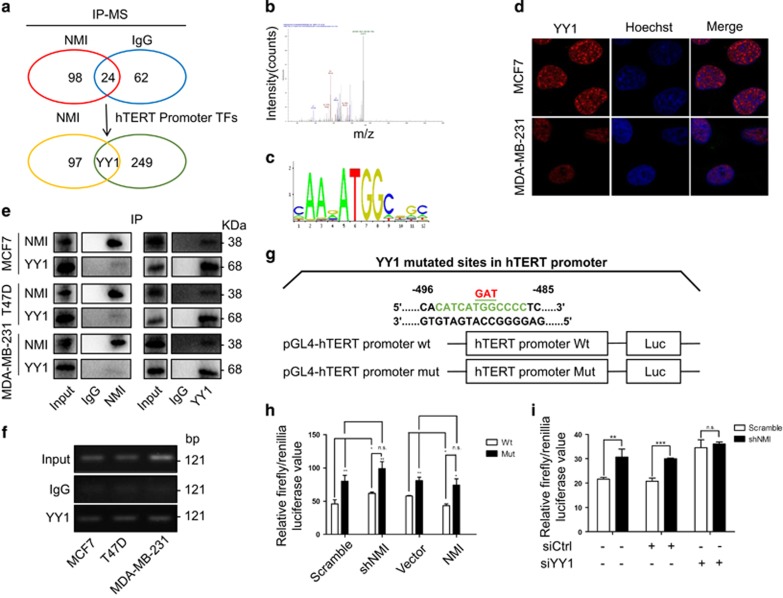
YY1 interacts with NMI to mediate the downregulation of hTERT transcription. (**a**) Mass spectrometry data of YY1. (**b**) The sequence used to predict YY1 binding site. (**c**) YY1 was predicted as the NMI binding proteins using the data from mass spectrometry analysis of the NMI-immunoprecipitated proteins and the data from the hTERT promoter region transcription binding site analysis. (**d**) MCF7 and MDA-MB-231 cells grown on chamber slides were cultured for 48 h, and the subcellular localization of YY1 was examined by confocal microscopy analysis with a confocal microscope. (**e**) The nuclear extracts of human breast cancer cells were prepared for immunoprecipitation using an antibody against NMI or YY1 and then evaluated by western blot. (**f**) ChIP analyses of YY1's binding on hTERT promoter. (**g**) The YY1 binding site-mutated hTERT promoter luciferase plasmids were constructed. (**h**) MCF7 cells were transfected with the plasmids of the hTERT promoter-driven luciferase with YY1 binding site mutation for 48 h followed by a dual luciferase assay. (**i**) MCF7 cells were co-transfected with shNMI and siYY1 or negative control siRNA, and then the cells were transfected with the hTERT promoter-driven luciferase plasmid. After 24 h, the promoter activity was analyzed. The data were represented as the mean±S.D. of three independent experiments. The level of significance was indicated by *P<0.05, **P<0.01, ***P<0.001

To determine whether the downregulation of hTERT expression mediated by NMI was realized by cooperating with some other transcriptional factors similarly binding to hTERT promoter, we used co-immunoprecipitation to pull down the potential proteins which interact with NMI protein. Mass spectrometry (MS) analysis identified 98 proteins in the cell nuclear protein extracts specifically interacting with NMI, but not with IgG (Figure 6a). We also predicted multiple potential transcription factor binding sites in the hTERT promoter region (+40 to −902) by transcription factor prediction software,^35^ and 250 proteins were identified with the potential of binding to hTERT promoter. Among all these proteins, YY1 was discovered as a new candidate which could not only bind to the hTERT promoter but also interact with the NMI protein (Figure 6a). The mass spectra of YY1 and its predicted binding site on hTERT promoter was respectively shown in Figures 6b and c. YY1 is a well-known transcription factor for its dual roles in regulating gene expression, as an activator or a repressor, depending upon the context in which YY1 binds to.^36, 37, 38, 39, 40^ Next, we analyzed the subcellular localization of YY1 by immunofluorescent imaging and found that YY1 mainly localized in cell nucleus (Figure 6d), which was consistent with the localization of NMI in cell nucleus (Supplementary Figure 2). Co-immunoprecipitation assay also confirmed the interaction between of NMI and YY1 in different breast cancer cells (Figure 6e). To further verify that YY1 binds to the hTERT promoter specifically in breast cancer, we also performed chromatin immunoprecipitation assay to detect the binding ability of YY1 to the endogenous hTERT promoter. As we expected, more chromatin hTERT promoter DNA were amplified with YY1 antibody than IgG itself (Figure 6f). As YY1 binding site (CATCATGGCCCC) in hTERT promoter region was predicted, we made a mutation within the site (Figure 6g). To identify whether the mutated site could influence hTERT promoter-driven luciferase activity, we co-transfected MCF7 cells with NMI-specific shRNAs or its overexpressing plasmids and pGL4-hTERT promoter luciferase plasmids (WT or Mut), and then analyzed the activity of luciferase reporter. The results showed that when YY1 binding site was mutated, the NMI-mediated downregulation of hTERT promoter-driven luciferase expression was blocked and the upregulation of hTERT promoter-driven luciferase expression caused by NMI knockdown was also attenuated (Figure 6h). In agreement with this, when YY1 was knocked down, the NMI silencing-mediated upregulation of hTERT promoter-driven luciferase expression showed no difference with control group (Figure 6i). These data together suggest that NMI interacts with YY1 to mediate the downregulation of hTERT transcription in breast cancer.

### NMI inversely correlates with hTERT in breast cancer samples

**Figure 7:**
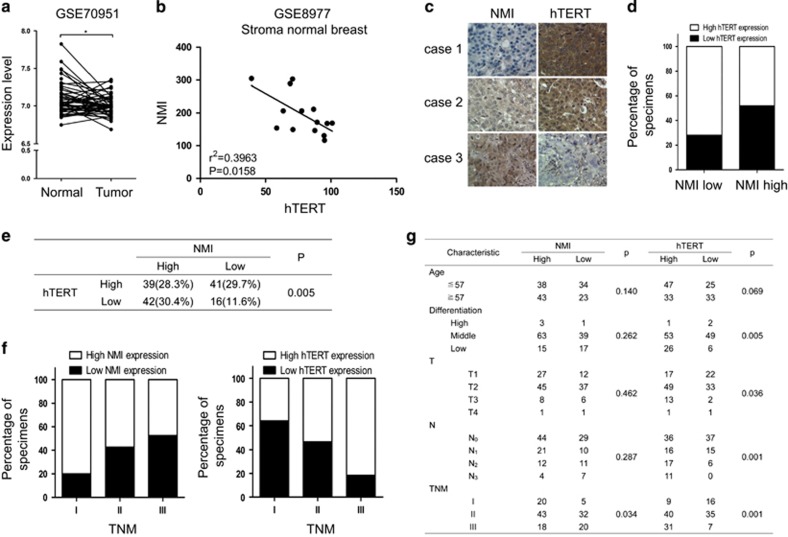
NMI inversely correlates with hTERT in breast cancer samples. (**a**) NMI mRNA expression level was detected using GEO database (GSE70951) from 43 pairs of human breast normal and corresponding tumor tissue. (**b**) The expression level of NMI and hTERT was detected using genome mRNA expression profiling data (GSE8977) from GEO database. (**c**) The representative images for NMI and hTERT by IHC analysis in 138 human breast cancer specimens, (X 200). (**d**) Percentages of specimens with either low or high NMI expression relative to hTERT level (*P*=0.005). (**e**) The correlation of NMI with hTERT in breast cancer tissues was examined. Significance of correlation between NMI and hTERT was determined by using *χ*^2^ test. (**f**) NMI expression level was negatively correlated with the worse TNM stage (*P*=0.034), and hTERT expression level was positively correlated with the worse TNM stage (*P*=0.001). (**g**) The relationship between the clinical characteristics and NMI/hTERT expression

Next, we studied the clinical significance of NMI-hTERT axis in patients with breast cancer. We first analyzed NMI level in paired human normal breast tissue and corresponding breast tumor tissue using GEO database (GSE70951). The decreased expression of NMI in tumor tissue compared with normal tissue was shown (Figure 7a). To investigate the correlation of NMI and hTERT, we analyzed the data of stroma normal breast samples from GEO database (GSE8977). It was shown that NMI expression was inversely correlated with hTERT expression (Figure 7b). We further assessed the expression of NMI and hTERT in breast carcinomas from 138 cases by immunohistochemical staining (Figure 7c). We also found the inverse correlation between NMI and hTERT expression in these tumor tissue samples (Figures 7d and e). Moreover, the high NMI expression but low hTERT expression was shown to be significantly correlated with the better status of clinical TNM stages, while the low NMI expression but high hTERT expression predicted the worse status of clinical TNM stages in breast cancer patients (Figures 7f and g). Overall, these data demonstrated again the inverse regulation of hTERT expression by NMI and their prognostic significance in breast cancer.

## Discussion

Recent studies have demonstrated that NMI is associated with DNA damage, cell cycle control and epithelial–mesenchymal transition (EMT). Previous studies showed that loss of NMI in breast cancer progression could be one of the driving factors that enhance invasive ability of breast cancer by aberrant activation of TGF-*β*/SMAD signaling. Our experiment also verified NMI’s influence on the invasion ability in breast cancer and found another pathway involved. However, the functions of NMI and its underlying molecular mechanisms as a tumor suppressor in breast cancer stem cells (BCSCs) remain unclear. In our study, we have revealed the function of NMI in weakening CSCs traits in BCSCs and illustrated the underlying molecular mechanisms of such function. NMI knockdown promotes CSCs expansion and tumorigenicity of breast cancer cells, while NMI overexpression inhibits CSCs properties. Moreover, we have shown that NMI suppresses hTERT expression, thereby inhibiting cancer stem-like cell traits in breast cancer *in vitro* and *in vivo*. Especially in the lung metastasis model of nude mouse, NMI overexpression significantly inhibits lung metastasis ability of breast cancer cells, and furthermore, the limiting dilution tumor formation experiments indicated only the mouse injected with the highest concentration of breast cancer cells with NMI overexpression formed tumors,fully demonstrating the decreased stemness-like capacity in breast cancer cells with NMI overexpression. Furthermore, YY1 was identified to functionalize as the synergistic effector of NMI to co-regulate hTERT transcription. To the best of our knowledge, it might be the first time to report that NMI targets hTERT via cooperation with YY1 to inhibit cancer stem-like cell traits and tumor growth in breast cancer. All the data might serve as a basis for discovering and identifying the novel therapeutic targets for breast cancers.

Cancer stem cells possess the capacity to both self-renew and differentiate.^41, 42^ Telomerase is critical for the integrity of stem cell compartments. In cancer stem cells, telomerase is more efficient at telomere maintenance rather than being reactivated. Thus, a better understanding of these molecular events will help refine approaches to targeting telomerase in cancer stem cells. As the core component of telomerase, hTERT can promote cancer metastasis and recurrence, and is closely correlated with aggressiveness and poor prognosis of many different human carcinomas. The direct regulation of hTERT on BCSCs traits was not represented in our study, however, its transcription and expression was found to be strictly controlled by NMI in breast cancer cells. Previous studies showed that the complex of BRCA1, c-Myc and NMI impaired induction of hTERT promoter activity mediated by c-Myc. In this study, we identified the influence of the NMI-hTERT axis on BCSCs, and also revealed the involvement of YY1 in the regulation of hTERT expression mediated by NMI. Not only that, hTERT knockdown or overexpression significantly reversed NMI knockdown or overexpression-mediated promotion or suppression in BCSCs stemness and tumor development, fully clarifying hTERT functionalized as the key downstream target molecule in mediating NMI’s tumor-suppressing effect in breast cancer progression, and also indicating the indispensable role of hTERT in the maintenance of BCSCs traits. NMI/hTERT signaling inhibition may be a striking target for potential CSCs therapeutics in breast cancer.

Here, we show that NMI knockdown notably upregulated hTERT promoter-driven luciferase expression and thereby upregulated hTERT expression, implicating the reverse transcriptional regulation of hTERT by NMI. Given the fact that NMI acts as a common transcriptional co-activator, and NMI lacks DNA-binding domains and is involved in the transcriptional regulation via recruiting and cooperating with many different sequence-specific transcriptional factors to indirectly anchor at gene promote regions,^8^ we deduce there must be some other transcriptional factors co-anchoring at hTERT promoter regions with NMI and contributing their supporting and synergistic function in such reverse transcriptional regulation. Based on IP and MS identification and software prediction of possible hTERT promoter-binding proteins, YY1 was focused and its involvement in NMI-mediated transcriptional regulation of hTERT was confirmed, as evidenced by the site mutation within its binding sequence at hTERT promoter blocked NMI expression change-mediated hTERT promoter activity change, and YY1 silencing consistently changed NMI knockdown-mediated hTERT promoter activity enhancement. Hence, most likely, NMI cooperates with YY1 to downregulate hTERT transcription and expression and thereby inhibit the stemness and growth of breast cancer cells. Besides YY1, some other transcriptional factors may also contribute to such regulation. What are these factors? How do they functionalize in NMI-YY1-hTERT axis-mediated reduction of BCSCs stemness? If it is irreplaceable, does it play positive or negative regulatory effect? All these questions deserve better investigation in our further study.

**Figure 8:**
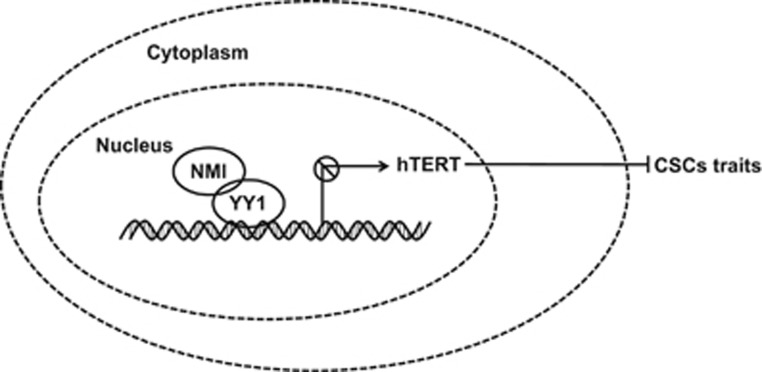
A schematic model of the role of NMI in regulating CSCs traits. NMI-YY1 component binds to hTERT promoter to inhibit hTERT transcription, thereby inhibiting cancer stem-like cell traits in breast cancer

In summary, we found that NMI inhibited cancer stem cell traits in breast cancer by downregulating hTERT signaling. We also showed that NMI was lowly expressed in breast cancer stem cell-enriched populations and the expression of NMI was negatively correlated with hTERT in breast cancer. The low NMI but high hTERT expression predicted the worse status of clinical TNM stages in breast cancer patients. Further mechanistic insights demonstrated that NMI functioned to regulate hTERT through its interaction with YY1 in breast cancers. Based on the data from our study, we propose a working model of NMI in the regulation of BCSCs (Figure 8). Taken together, our results demonstrated that NMI repressed breast cancer stem cell traits by downregulating hTERT, and suggest that the NMI-hTERT signaling axis might provide a novel therapeutic target for the inhibition of breast cancer progression and metastasis.

## Materials and methods

### Cell culture and stable cell line selection

Human breast cancer MCF7 were grown in RPMI 1640 media supplemented with 10% FBS. T47D were grown in RPMI 1640 media supplemented with 10% FBS and 0.2 units/ml insulin. MDA-MB-231 was grown in DMEM media supplemented with 10% FBS. All cells were grown in a 37 °C humidified incubator with 5% CO_2_. Stable silencing of NMI expression was accomplished using short-hairpin RNA cloned into psi-LVRH1GP vector (GeneCopoeia, Rockville, USA). The vector containing a non-targeting shRNA was used as a control. NMI overexpression was accomplished by cloning NMI cDNA into pEZ-Lv203 (GeneCopoeia), and the empty vector was used as a control. The vectors were transfected using Lenti-Pac HIV Expression Packaging Kit (GeneCopoeia, Cat: HPK-LvTR-20). The cells were selected in media supplemented with 5 *μ*g/ml of puromycin.

### Mammosphere culture

Cells were cultured in DMEM/F-12 supplemented with 2% B27, 20 ng/*μ*l EGF and 10 ng/*μ*l bFGF. Two thousand cells were seeded in 35 mm ultra-low attachment plate. After 2 weeks of culture, the spheres with diameters larger than 50 *μ*m were counted. When mammospheres were passaged, sphere pellets were centrifuged and digested with trypsin-EDTA.

### qRT-PCR

Total RNA was extracted using Trizol reagent (TaKaRa, Dalian, China) and was transcribed into cDNA using the EasyScript One-Step gDNA Removal and cDNA Synthesis SuperMix (Transgen, Beijing, China, Cat: AE311). The expression levels of the mRNAs were determined by qRT-PCR using SYBR Green (TaKaRa) in an Mx3005 real-time PCR detection system. ATCB was used as an internal control. All data represent the average of three repeated experiments. The primers used were listed in Supplementary Table 1.

### Flow cytometry

The identification of CD44^+^CD24^-^cells was performed using APC mouse anti-human CD44 (BD Bioscience, CA, USA, Cat: 559942) and PE mouse anti-human CD24 (BD Bioscience, Cat: 555428) antibodies. The APC mouse IgG2b K Isotype control (BD Bioscience, Cat: 555745) and the PE mouse IgG2a K Isotype control (BD Bioscience, Cat: 555574) were used as the isotype controls. Cells were analyzed using an Accuri C6 Flow Cytometer.

### Immunohistochemistry

Tissue microarray sides containing 138 breast carcinomas were purchased from Shanghai Outdo Biotech (Shanghai, China). The slides were heated at 65 °C for 30 min, followed by paraffin removal with xylene and subsequent rehydration with ethanol. Antigen retrieval was performed in a chamber containing citrate buffer (pH 6.0) for 20 min maintaining at a sub-boiling temperature. Samples were blocked with 10% goat serum for 1 h each at room temperature. 200 *μ*l primary antibody was added to each slide and incubated overnight at 4 °C (NMI monoclonal, Santa Cruz, Texas, USA, 1:100; hTERT monoclonal, Santa Cruz, 1:100). The visualized signal was developed with 3, 3′-diaminobenzidine (DAB) and the slides were counterstained in hematoxylin. The immunostaining analysis of NMI and hTERT protein expression was done based on these tissue microarrays. For each tissue sample, protein expression was scored according to the staining color: negative staining (no yellow); low staining (light yellow); moderate or high staining (yellow brown or brown). With prior written consent from patients, all the tissue samples had been obtained before anti-cancer treatment.

### Transwell invasion assay

Cell invasion ability was detected using 24-well chemotaxis chambers (Corning, CA, USA, Cat: 3422). The cells were washed twice with PBS, resuspended in 100 *μ*l serum-free medium and added into the upper chambers. The lower chambers were filled with 500 *μ*l medium containing 20% fetal bovine serum (FBS). The cells were incubated for 36/60 h in the upper chamber coated with a mixture of serum-free medium and Matrigel (4:1; BD Biosciences, Cat: 356234). The membrane were fixed in methyl alcohol for 10 min at room temperature, stained with crystal violet for 10 min, washed 3 times with PBS and dried off. The crystal violet was dissolved with 500 *μ*l 33% acetic acid and the OD570 value was measured.

### Dual luciferase assays

The hTERT promoter (−902 to +40) was amplified using normal human DNA as a template and cloned into the pGL4-Basic (Promega, Beijing, China). MCF7 cells were seeded in 6-well plates. On the next day, the cells were transfected with NMI sh-plasmids or control plasmid. At 24 h after transfection, the cells were transfected with hTERT reporter plasmid and a pRL-TK internal control. At 48 h after transfection, luciferase activity was measured using the dual luciferase reporter assay system (Promega, Cat: E1910).

### Co-immunoprecipitation assays

Nuclear protein lysates were first incubated with anti-NMI antibody or mouse IgG overnight at 4 °C under rotation, and then incubated with protein A/G plus-agarose beads (Santa Cruz Biotechnology, Texas, USA, Cat: sc-2003) for 2 h. The precipitates were washed with 500 *μ*l PBS for three times, resuspended in 50 *μ*l 2 × loading buffer, boiled at 95 °C for 10 min and subjected to western blot analysis.

### Chromatin immunoprecipitation assays

The cells were fixed with 1% formaldehyde for 10 min at 37 °C, then sonicated on ice to shear the DNA to 200 to 500 bp. One-third of the total cell lysate was used as the DNA input control. The other two thirds of the lysate were subjected to immunoprecipitations with anti-YY1 and IgG antibody. The DNA was subjected to PCR to amplify a 121 bp region of the hTERT promoter. The PCR products were resolved electrophoretic ally on a 2% agarose gel and visualized by ethidium bromide staining. The primers used were listed in Supplementary Table 2.

### Immunofluorescence and confocal microscopy

The cells were washed with PBS and were fixed with 4% paraformaldehyde (w/v) for 30 min and permeabilized with 0.2% (w/v) Tritonx-100 in PBS for 5 min in dark. Then, the samples were blocked with 10% BSA for 1 h, and incubated with the primary antibody which were diluted in PBS containing 1% BSA overnight. After PBS washings, cells were incubated for two hours with secondary antibody conjugated with Alexa Fluor 488 or Alexa Fluor 555. The nuclei were stained with Hoechst 33342.

### Animal studies

All animal experiments were performed according to the Dalian Medical University for the Care and Use of Laboratory Animals. Female Balb/c nude mice at the age of 4 to 6 weeks were used in all studies. Starting from one week before MCF7 cancer cell line injection, the mice were treated with subcutaneous injection of estradiol cypionate (2 mg/kg in corn oil, MedChemExpress, Shanghai, China, Cat: HY-B1100) every week for 4 weeks in tumorigenesis assays. The GFP-labeled MCF7 cells (3 × 10^6^ in 100 *μ*l PBS) were injected subcutaneously into the left flank of each mouse. One group was injected with the control shRNA GFP-labeled MCF7 stable cell line, the second group was injected with the NMI knockdown GFP-labeled MCF7 stable cell line, the third group was injected with the NMI knockdown/control vector GFP-labeled MCF7 stable cell line and the fourth group was injected with the NMI knockdown/hTERT-knockdown GFP-labeled MCF7 stable cell line. The tumor size was measured using Vernier calipers once every three days from the twelfth day after injected and volumes were calculated as following: *V*=(width^2^ × length)/2. At 21 day, the mice were narcotized and the tumors were detected by Bioluminescence. Tissues of tumor were fixed in 10% formalin and embedded in paraffin for histologic analysis. GFP-labeled MDA-MB-231 cells (1 × 10^6^ cells in 100 *μ*l PBS) were injected subcutaneously into the left flank of each mouse. One group was injected with the control vector GFP-labeled MDA-MB-231 stable cell line and the second group was injected with the NMI overexpression GFP-labeled MDA-MB-231 stable cell line. To identify cell metastasis ability, the GFP-labeled MDA-MB-231 stable cell lines were injected into tail vein and the lung metastasis ability were detected by H&E staining and bioluminescence using fluorescence microscope.

### Statistical analyses

The data are presented as mean±S.D. in the figures. A Student *t*-test was performed to compare the *in vitro* data. A log-rank test was performed to compare tumor-free survival. *P*-values less than 0.05 were considered statistically significant.
