# Supplementary figures and images for: NMI inhibits cancer stem cell traits by downregulating hTERT in breast cancer
Source: Cell Death Dis. 2017 May 11;8(5):e2783–. doi: 10.1038/cddis.2017.200 (PMC5520720; doi:10.1038/cddis.2017.200)

Supplementary Figure 1

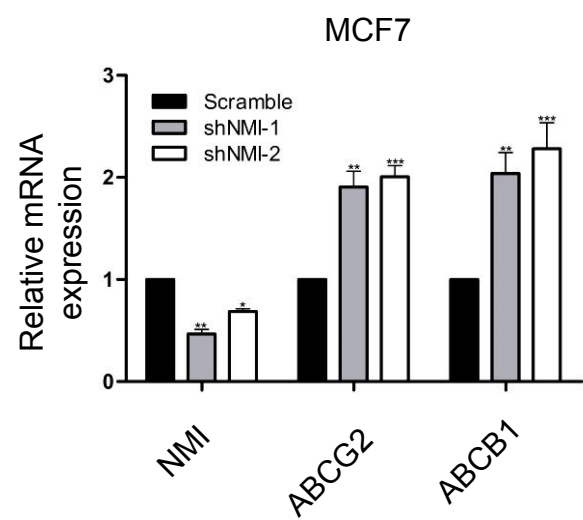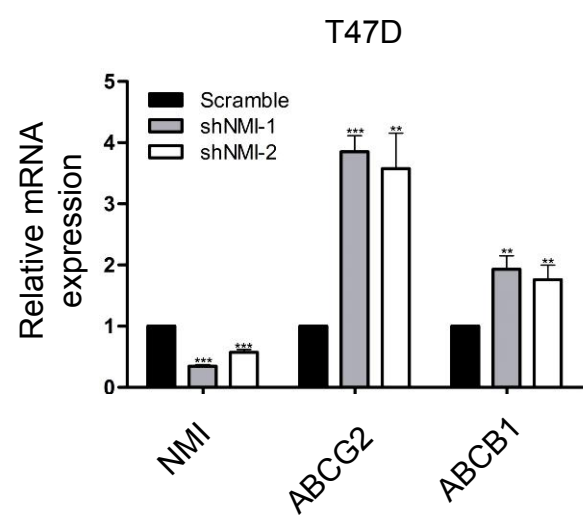

Supplementary Figure 2

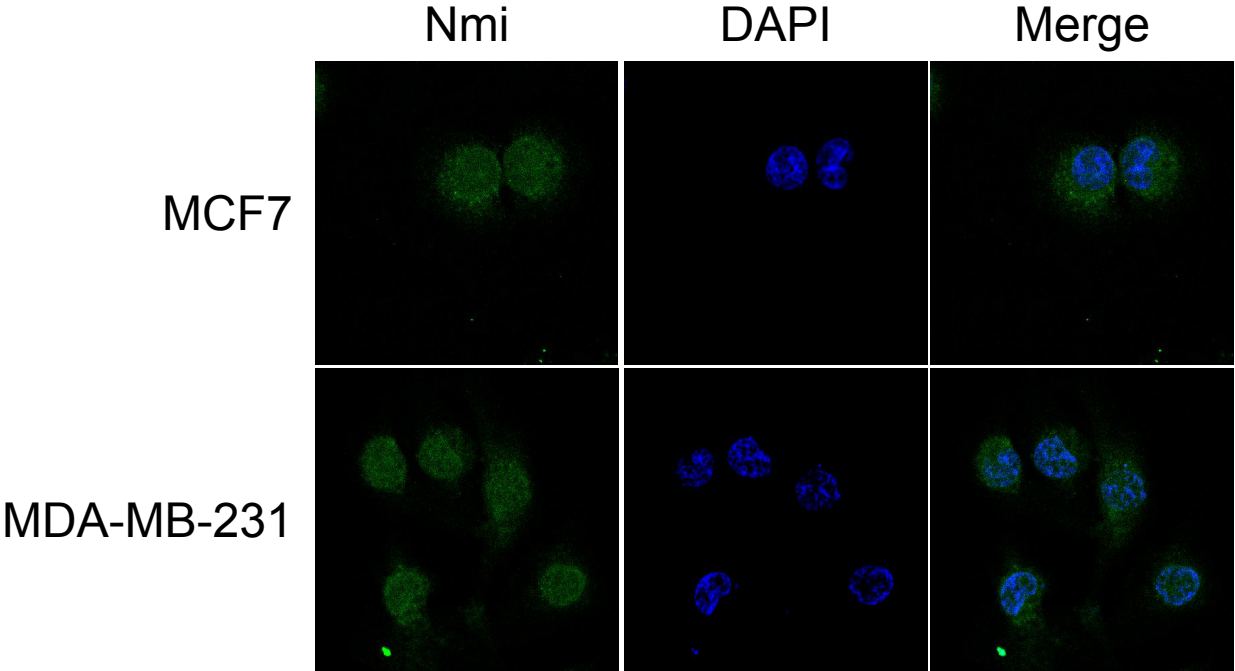

Supplement: Supplementary Figures [file cddis2017200x1.pdf]
