# Supplementary material for: NMI inhibits cancer stem cell traits by downregulating hTERT in breast cancer
Source: Cell Death Dis. 2017 May 11;8(5):e2783–. doi: 10.1038/cddis.2017.200 (PMC5520720; doi:10.1038/cddis.2017.200)
Supplement: Supplementary Tables [file cddis2017200x2.doc]

**Table 1.** The primer sequences for qRT-PCR

| **Gene** | **Forward Sequence (5’-3’)** | **Reverse Sequence (5’-3’)** |
| --- | --- | --- |
| NMI | AAGGAGCATTCGCCAGATGAA | GTAGCCTCTTGTAACTCCGTTTC |
| NANOG | TTTGTGGGCCTGAAGAAAACT | AGGGCTGTCCTGAATAAGCAG |
| OCT4 | GTGTTCAGCCAAAAGACCATCT | GGCCTGCATGAGGGTTTCT |
| SOX2 | GCCGAGTGGAAACTTTTGTCG | GGCAGCGTGTACTTATCCTTCT |
| β-actin | GGCACCCAGCACAATGAA | TAGAAGCATTTGCGGTGG |

**Table 2.** The primer sequences for ChIP

| **Gene** | **Forward Sequence (5’-3’)** | **Reverse Sequence (5’-3’)** |
| --- | --- | --- |
| hTERT promoter | GTCTCCGGATCAGGCCAGC | CCAGCGGAGAGAGGTCGAAT |
